# Discretized Theta-Rhythm Perception Revealed by Moving Stimuli

**DOI:** 10.1038/s41598-018-24131-6

**Published:** 2018-04-09

**Authors:** Ryohei Nakayama, Isamu Motoyoshi, Takao Sato

**Affiliations:** 1 Department of Life Sciences, The University of Tokyo, 3-8-1 Komaba, Meguro-ku, Tokyo, 153-8902 Japan; 2 Department of Comprehensive Psychology, Ritsumeikan University, 2-150 Iwakura-cho, Ibaraki, Osaka, 567-8570 Japan

## Abstract

Despite the subjective continuity of perception over time, increasing evidence suggests that the human nervous system samples sensory information periodically, a finding strongly exemplified by discretized perception in the alpha-rhythm frequency band. More recently, studies have revealed a theta-band cyclic process that manifests itself as periodical fluctuations in behavioral performance. Here, we used a simple stimulus to demonstrate that the theta-cyclic system can produce a vivid experience of slow discrete visual sampling: a Gabor texture pattern appears as a series of flickering snapshots if its spatial window moves continuously over a carrier grating that remains still or drifts continuously in the opposite direction. While the perceptual magnitude of this illusory saltation varied with the speed difference between grating and window components in head-centered coordinates, the perceived rhythm of saltation remained nearly constant (3–8 Hz) over a wide range of stimulus parameters. Results provide further evidence that the slow cyclic neural processes play a critical role not only in attentional task performance but also in conscious perception.

## Introduction

Increasing evidence suggests that the human brain processes sensory information in a periodic manner^1–3^. At the behavioral level, the distributions of reaction times and correct detections for visual targets often exhibit a periodicity near 10 Hz^4–6^. At the physiological level, EEG and MEG studies demonstrate that the perception of motion, grouping, and causality between successive stimuli depends on the peri-stimulus phase of oscillatory cortical activity in the alpha band (8–15 Hz)^7–10^. It is generally believed that cyclic neural processing plays a significant role in the integration of temporally diverse visual information into a single perceptual moment.

In favor of this argument, several studies report illusions in which a continuously changing stimulus is perceived as a series of discrete snapshots (“discrete perception” in our definition), as is perhaps best exemplified by a chromatic line that appears to jitter at a temporal rate of 10–11 Hz when embedded in a luminance-defined moving target^11,12^. Similarly discretized perception has been reported for dynamic visual noise, radial patterns, and LSD-induced visual trails^13–15^. These illusory phenomena provide evidence for a direct link between cyclic processing and conscious perception, and they intimate that temporal continuity in visual perception, rather than passively mirroring continuous image inputs, is actively constructed by specific neural mechanisms.

More recently, several studies imply the existence of another cyclic process operating at slower temporal rates than alpha rhythms. Psychophysical studies show that behavioral performance for contrast detection and temporal segregation fluctuates around 3–8 Hz relative to the timing of a visual cue or the observer’s action^16–20^. Similarly, the spatiotemporal localization of moving stimuli depends on the phase of theta-band oscillations in parietal and occipital cortex^21^. In contrast to discretized alpha-rhythm perception^11–15^, discrete theta-rhythm perception has not yet been reported. Slow cyclic processes have been inferred from periodical fluctuations in behavioral performance across trials^16–22^, and this suggests that theta rhythms originate from post-perceptual, or decisional, levels rather than from perceptual levels.

**Figure 1:**
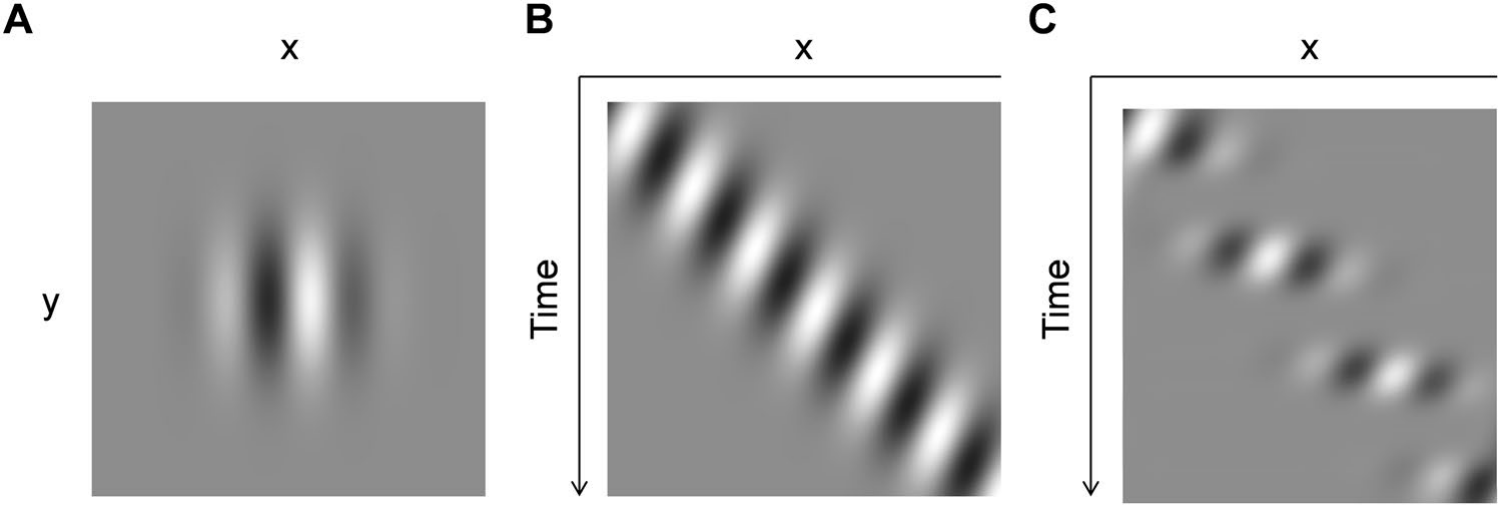
Visual stimulus and illusory saltation. (**A**) Observers view a conventional Gabor grating (x-y plot). (**B**) Spatial window moves continuously in space whereas the carrier grating drifts continuously in the opposite direction (x-t plot). (**C**) Subjective percept (illusory saltation): the whole stimulus appears to move intermittently and jumps in discrete steps (x-t plot; see S. I. Movie S1 for demonstrations).

Here, we use a simple display demonstrating an illusion whereby the visual perception of continuously moving stimuli is discretized in the theta frequency band. Consider a visual Gabor pattern whose window changes location smoothly from left to right while its underlying carrier grating moves in the opposite direction (Fig. 1A,B). For such stimuli, the whole stimulus is perceived as a slow succession of discontinuous stationary snapshots (Fig. 1C). The present study shows that illusory saltation is clearly observed if the carrier grating remains stationary or drifts in the direction opposite to that of the blurry spatial window. Critically, the magnitude of the illusion varies lawfully only if relative velocity is expressed in head-centered coordinates (i.e., the reference frame in which spatial attention operates) instead of retinotopic coordinates (Experiment 1). We found that the perceived rhythm of saltation remained nearly constant (3–8 Hz) over a wide range of stimulus parameters (Experiments 2 and 3). We take this as further evidence of the involvement of the theta-cyclic system in the conscious perception.

## Experiment 1

In order to examine what stimulus parameters determine the percept of illusory saltation, we presented various moving stimuli and had observers judge the subjective strength of the illusion. We manipulated the speed of the spatial window, of the carrier grating, and of the tracking fixation.

### Method

#### Observers

Four naive participants and one of the authors (RN) participated in Experiment 1. Two of the naive participants and RN participated in Experiments 2 and 3. All participants had normal or corrected-to-normal vision. All the experiments were conducted in accordance with the Declaration of Helsinki and approved by the ethics committee at the University of Tokyo. All participants provided written informed consent.

#### Apparatus

All experiments were conducted in a dark room. Images were displayed on a gamma-corrected 21-inch CRT (Mitsubishi Diamondtron M2 RDF223G; 800 × 600 pixel) through a video attenuator (Bits++, Cambridge Research Systems Ltd.) with a frame rate of 150 Hz. The pixel resolution of the CRT was 3.0 min/pixel at a viewing distance of 57 cm and the mean luminance was 49.3 cd/m^2^. Throughout the experiment, movements of both eyes were monitored by means of Viewpoint Eye Tracker (220 Hz; Arrington Research, Inc.).

#### Stimuli

Visual stimuli consisted of a vertical sinusoidal grating pattern spatially windowed by a Gaussian function (Gabor grating). The carrier grating and its spatial window moved horizontally at one of several velocities. The standard deviation of the Gaussian window was 1 deg, carrier spatial frequency was 0.5 cycle/deg, and luminance contrast was 0.2. The initial spatial phase angle of the grating was randomized and the direction of the moving window was alternated across trials. A fixation point (0.8 deg in diameter, 98.6 cd/m^2^) either remained stationary in the center of the screen or moved horizontally with a vertical 8 deg spatial offset relative to the motion trajectory of the window. On each trial, after 1-sec fixation period, stimuli gradually appeared and disappeared against a gray background according to a half-cycle sinusoidal contrast-ramping temporal profile of 2 sec.

#### Procedures

**Figure 2:**
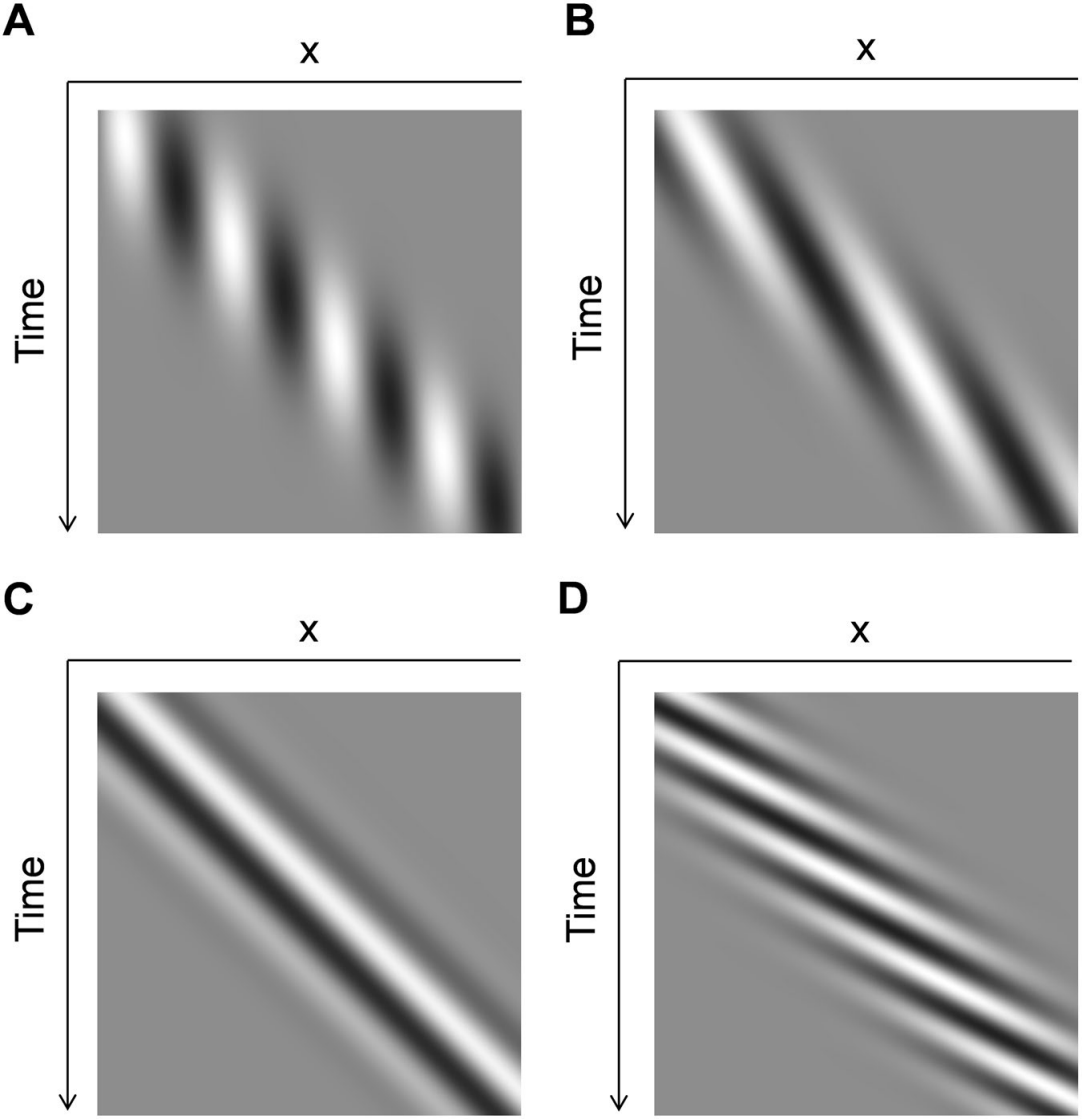
Examples of experimental stimuli. (**A**) A spatial window moves continuously while the carrier grating remains stationary (x-t plot; see S. I. Movie S2). (**B**,**C**,**D**) A carrier grating drifts in the same direction as a spatial window. The carrier moves more slowly (**B**), at the same speed (**C**), and more quickly (**D**) compared to the window’s speed (x-t plot; see S. I. Movies S3, S4, and S5 respectively). Note that panels (A) and (B) correspond to “negative” grating speeds relative to window position on the abscissa of Fig. 4A while (**C**) and (**D**) correspond to “0 deg/sec” and “positive” relative speeds respectively.

**Figure 3:**
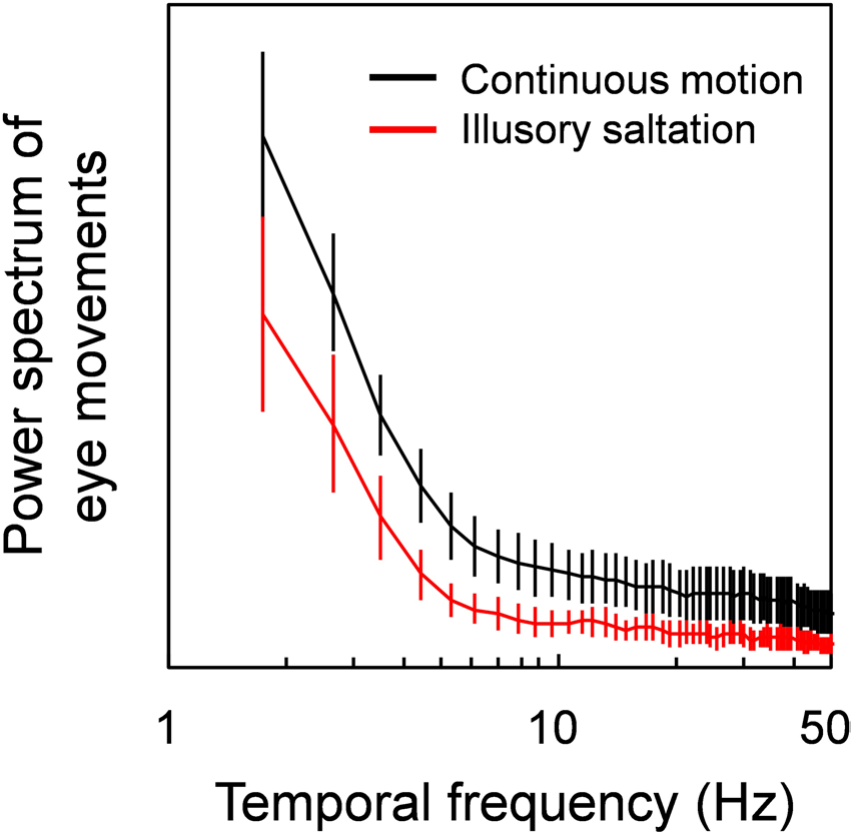
Power spectrum of eye movements. Curves show the average power spectra of eye movements fixation for trials that elicited illusory saltations (red) and continuous motion (black). Error bars are +− 1 SE.

Observers were asked to gaze at the fixation point and report the subjective magnitude of illusory saltation using a nine-point scale (for instance, “0” if no saltation and only continuous motion was perceived, “4” if saltation occurred in about half the period of stimulus presentation, or “8” if it was perceived as moving only in discrete steps). Window velocity varied from 0 to 16 deg/sec and grating velocity varied from −40 to 16 deg/sec relative to the window (negative velocity indicates that the grating moved in the opposite direction from the window; see Fig. 2). Various combinations of grating and window velocities were interleaved within each experimental block. To address the possibility that fixation eye movements (i.e. micro-saccades) influenced the percept of illusory saltation, we computed each trial’s eye movements power spectrum over a one-second window (trial period from 0.5 to 1.5 sec) and compared spectra between illusory-saltation (observer rating > 5) and continuous-motion trials (observer rating < 3) from 1.8 to 49.5 Hz. Power was not significantly different between illusory-saltation and continuous-motion trials (Fig. 3; ANOVA: *F*_(1, 4)_ = 5.536, *p* = 0.078, *n.s*.) and, if anything, power for continuous-motion trials tended to be somewhat larger than illusory-saltation trials. From this we conclude that eye movements should not play a major role in the percept of illusory saltation.

In the tracking condition, observers tracked a fixation point that moved at 8 deg/sec in the same direction as the window motion. To facilitate the tracking of a moving fixation point, fixation disappeared prior to its initial motion and reappeared at a position shifted by 1.2 deg toward the direction opposite to tracking before the trial continued on (step ramp method)^23^. The velocities of the grating and window components varied in the same manner as above. That is, in conditions where the window and the fixation point moved in tandem, the window moved in head-centered coordinates but remained still in retinal coordinates. Conversely, window velocity was 0 deg/sec when it moved only retinal coordinates. We verified the accuracy of fixation tracking for every trial by analyzing eye-tracking data during the trial period from 0.5 to 1.5 sec. The trajectory of the fixation point was first estimated by separately fitting a linear function to each eye’s positional data. A root mean square error (RMSE) in X-Y position was calculated between gaze and the estimated fixation point. This error was taken as a measure of the accuracy of tracking. We discarded trials in which the RMSE exceeded the 99% confidence interval of the average RMSE for a randomly chosen block of trials in the fixation condition for each observer. As a result, 90% (3490/3873) of trials were used in the subsequent analysis.

### Results

**Figure 4:**
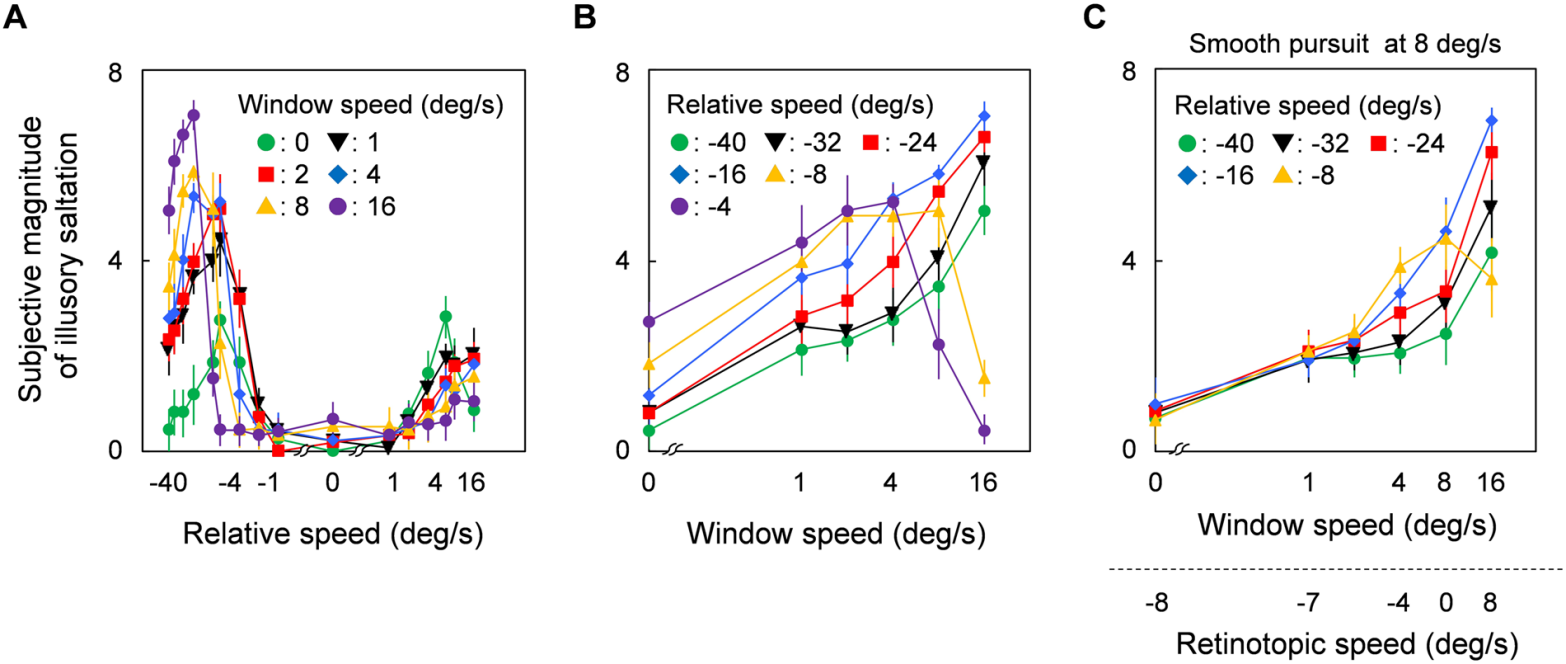
Apparent magnitude of illusory saltation averaged across observers. Error bars are +− 1 SE. (**A**) Subjective magnitude is plotted as a function of the relative velocity of the grating with respect to the window. Each color symbol corresponds to different window velocities. (**B**) The panel shows the same data as (**A**) but plotted as a function of window velocity. Each color symbol corresponds to different grating velocities relative to the window, parts of which were not plotted for simplicity. (**C**) The panel shows subjective illusion magnitude as observers tracked a fixation point moving at 8 deg/sec on a path parallel to the stimulus window. The two label rows below the abscissa denote window velocity in head-centered and retinotopic coordinates respectively. Grating velocities are the same as in (**B**).

Observers rated the subjective magnitude of illusory saltation for various combinations of velocities between carrier grating and spatial window. Figure 4A shows average ratings as a function of grating velocity relative to window position. Positive and negative velocities respectively indicate “same” and “opposite” directional drifts with respect to window motion. We found that opposite directional drifts elicited distinct percept of illusory saltation and that the range of grating speeds over which observers experienced illusory saltation depends on window speed. Figure 4B replots observer ratings as a function of window velocity. Ratings increase proportionally with window velocity up to the point where it exceeds absolute grating speed. For example, there is hardly any saltation for a window speed of 16 deg/sec and a grating drift of −8 deg/sec (yellow triangles) or for window speeds of 8 or 16 deg/sec and a grating drift of −4 deg/sec (purple circles): in these specific cases, opposite directional drift relative to window motion corresponds to the same directional motion on screen or retina. In short, illusory saltation occurs if the spatial window moves continuously over a carrier grating that remains stationary on screen or drifts in the opposite direction.

Although Fig. 4B shows that illusory saltation is a relatively lawful function of window speed if observers fixate a steady point on the screen, the data do not tease apart whether the effect depends on retinal or head-centered velocities. To disambiguate between the two possibilities, observers tracked a fixation point moving at 8 deg/sec along a path parallel to the window’s motion. If retinal motion is responsible for saltation, the illusion should disappear in the condition where the window also moves at 8 deg/sec on the screen (i.e., 0 deg/sec in retinotopic coordinates) while smooth eye pursuit maintains the window relatively stationary on the retina. Results show that observer ratings increase systematically with head-centered window velocity (Fig. 4C). These findings indicate that perception of illusory saltation is predominantly determined by head-centered window velocities rather than by retinal window velocities. Differences between smooth pursuit data (Fig. 4C) and steady fixation data (Fig. 4B) are presumably attributable to a minor contribution of retinal motion to the illusion. Additionally, our phenomenological investigations confirmed that no saltation occurred for stimuli with a luminance-defined (or sharp edged) window or with contrast-modulating grating carriers.

## Experiment 2

Results of Experiment 1 indicate that illusory saltation is a product of interactions (or conflicts) between luminance grating motion and second-order (contrast-defined) window position that involves higher-order motion signals defined in head-centered coordinates^11^. In this view, one would expect the temporal rate of illusory saltation to be a function of particular set of stimulus parameters. For example, the stimulus should appear to jump at a faster temporal rate as the window moves more rapidly. In order to identify stimulus components that determine dynamic appearance of saltation, we measured its apparent temporal frequency for various combinations of spatiotemporal frequencies and window sizes.

### Method

#### Stimuli

A Gabor grating was presented as in Experiment 1 and was subsequently followed by a 2-sec Gaussian blob (standard deviation = 1 deg) flickering on and off at a variable temporal frequency with 1-sec fixation periods between trials and stimuli. The luminance flicker was located at the center of the motion trajectory of the window. The luminance contrasts of the grating and the flicker were 0.8 and 1.0 respectively.

#### Procedures

Observers were asked to gaze at the fixation point and indicate a presence or absence of an illusory saltation percept and, if saltation was present, to report whether the temporal rate of the illusory was higher or lower than that of luminance flicker by pressing a button. The temporal frequency of the luminance flicker was manipulated according to an adaptive staircase method: the temporal frequency was increased/decreased if observers perceived the illusory saltation as faster/slower in the previous trial, or as the same as in previous trial if no saltation was perceived. Window velocity varied from 1.25 to 8 deg/sec and window size varied (as defined by its standard deviation) from 0.5 to 2 deg. Grating velocity varied from −20 to −1 deg/sec relative to the window (within the range where illusory saltation was reliably observed in Experiment 1) and spatial frequency varied from 0.5 to 4 cycle/deg. Various parameter combinations were interleaved within each experimental block. Stimulus combinations that provided no saltation in more than half of the trials were categorized as moving continuously (0 Hz apparent frequency). For the other stimulus combinations, we estimated the temporal frequency of luminance flicker corresponding to the point of subjective equality (i.e., the probability of perceptual judgments being divided equally between higher and lower temporal rate of flicker than saltation) using the maximum likelihood method^24–26^.

### Results

**Figure 5:**
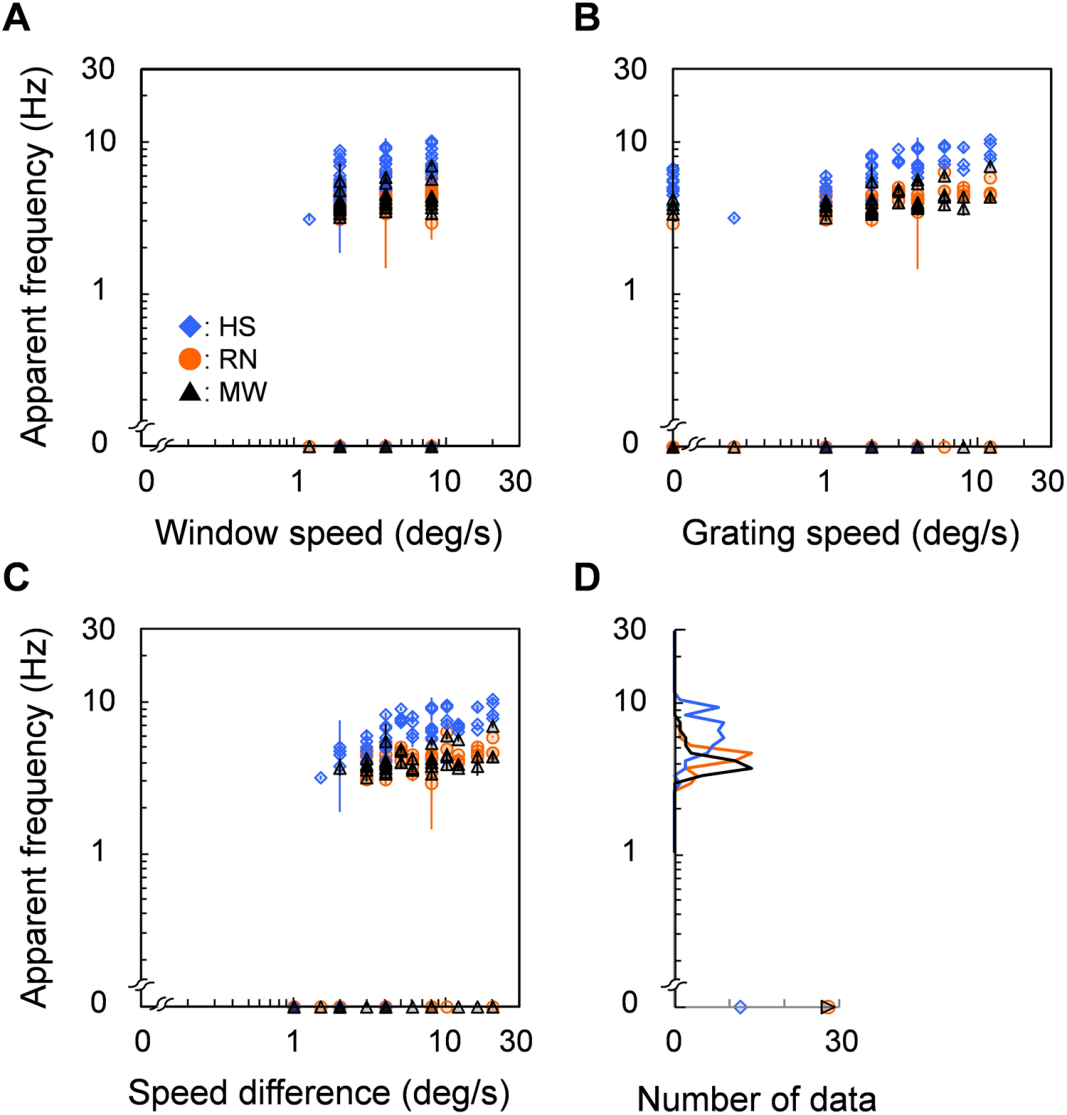
Results of matching measure. (**A**,**B**,**C**) Apparent temporal frequency of illusory saltation is plotted as a function of window speed (**A**), grating speed (**B**), and speed difference between them (**C**). Each color symbol corresponds to different observers. Error bars are +− 1 SE across 400 bootstrap samples. Stimuli that did not produce saltation (continuous motion percept) are denoted as 0 Hz. (**D**) Distribution of data counts binned according to apparent frequency (per 0.05 Hz in log scale).

Figure 5 plots apparent saltation frequency of as a function of window speed (A), grating speed (B), and speed difference between window and grating (C) on screen. Each color corresponds to different observers (n = 3). Rather than depend critically on specific combinations of stimulus parameters, most of the data (91%) were estimated to fall within a narrow 3–8 Hz band regardless of stimulus speeds for which saltation was observable. Next, we considered only trials in which saltation was observed and fitted a linear function separately to each observer’s data. We found slopes to be 0.16 (SE = 0.08), 0.21 (SE = 0.08), and 0.12 (SE = 0.05) against window speed, grating speed, and speed difference, respectively. All of them are not significantly larger than zero (t-test: *p* = 0.20, 0.12, and 0.15, *n.s*.). This tendency also holds as a function of stimulus size (slope = −0.07, SE = 0.23, t-test: *p* = 0.79, *n.s*.) and spatial frequency (slope = 0.14, SE = 0.04, t-test: *p* = 0.08, *n.s*.). Figure 5D reveals significantly narrower distributions of apparent-frequency data within each observer as well as sharper peaks except for HS. Peak frequency for HS is 5.96 Hz (SD = 1.72 Hz), 4.73 Hz (SD = 0.72 Hz) for RN, and 3.76 Hz (SD = 0.95 Hz) for MW excluding 0 Hz. We note individual differences in apparent saltation frequency. As evidenced by both group- and individual-based analyses, these results suggest that the temporal rate of illusory saltation depends linearly neither on stimulus speed, size, or spatial frequency.

## Experiment 3

Results of Experiment 2 reveal that, at a minimum, the perceived rhythm of saltation is not a simple function of particular set of stimulus parameters. In order to investigate the relation between them in greater detail, we re-measured the apparent temporal frequency of illusory saltation by means of directly counting the number of perceptual saltation cycles. Measurements were taken for stimuli of various grating speeds since the strongest dependency (i.e., the steepest slope) on stimulus parameters in Experiment 2 was related to grating speed.

### Method

#### Apparatus

Images were displayed on a gamma-corrected 27-inch LCD (BENQ XL2730Z; 2560 × 1440 pixel) with a frame rate of 144 Hz. This monitor is a successor to the BENQ XL2410T that has been proven to have sufficient temporal precision to display fast-changing stimuli common in visual psychophysics^27^. The LCD’s pixel resolution was 1.2 min/pixel at a viewing distance of 65 cm and mean luminance was 137.78 cd/m^2^.

#### Stimuli & Procedures

A Gabor grating was presented as in Experiment 1 with the exception that stimuli were presented immediately at their nominal contrast (step profile) for a full 2 sec. as opposed to contrast being ramped smoothly on and off (cosine profile). In addition, in several experimental blocks, stimulus motion underwent 3 to 15 stop-and-go “jumps” whereby grating and window moved in locked tandem over a distance of 17.6 deg. Observers directly counted the number of illusory or physical saltation and reported it by pressing one of several numbered buttons (“0” for continuous motion). Window velocity was either 2.9 or 8.6 deg/sec and window size (standard deviation) was either 0.6 or 1.2 deg. Grating velocity varied from −14.4. to −1.7 deg/sec relative to the window and grating spatial frequency was either 0.9 or 1.2 cycle/deg. Physical-saltation stimuli had a fixed standard deviation of 0.6 deg and spatial frequency of 1.2 cycle/deg. Various parameter combinations were interleaved within each experimental block. Stimulus combinations that provided no saltation in more than half of the trials were categorized as continuous motion (0 Hz in apparent frequency).

### Results

**Figure 6:**
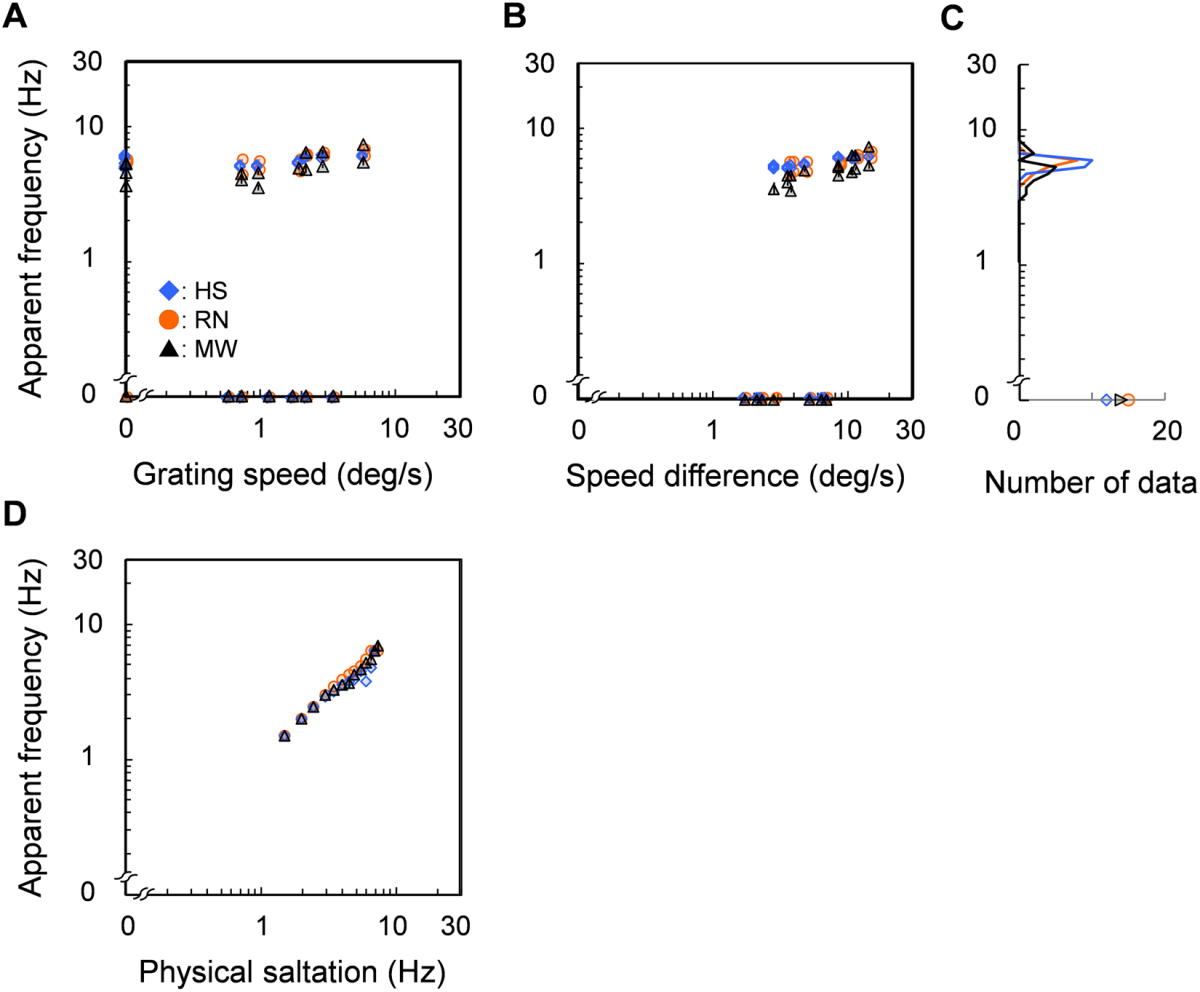
Results of counting measure. (**A**,**B**) Apparent temporal frequency of illusory is plotted as a function of grating speed (**A**), speed difference between window and grating (**B**). Each color symbol corresponds to different observers. Error bars are +− 1 SE across trial responses within observer. (**C**) Distribution of data counts binned according to apparent frequencies (per 0.05 Hz in log scale). (**D**) Apparent temporal frequency of physical saltation is plotted as a function of temporal frequency of physical saltation.

Figure 6 shows the apparent temporal frequency of saltation as a function of grating speed (A), speed difference between window and grating on screen (B), and actual temporal frequency of physical saltation (D). Each color corresponds to different observers (n = 3). As with the results of matching measure (Experiment 2), all the data fell within a narrow 3–8 Hz band regardless of stimulus speeds (Fig. 6A,B). Fitting a linear function to the data reveals mean slopes of 0.22 (SE = 0.06) and 0.15 (SE = 0.03) against grating speed and speed differences respectively. For these data, slope is not significantly larger than zero for grating speed alone (t-test: *p* = 0.07) whereas it is somewhat larger than zero for speed differences (t-test: *p* = 0.04). However, considering that apparent frequency varied only by a factor of 2× (3.5–7.3 Hz), or 1.6× on average for individual data, while physical speed difference varied by a factor of 5× (2.9–14.4 deg/sec), the functional relation between apparent frequency and speed difference is considerably weak. Figure 6C also reveals an extremely narrow distribution of apparent-frequency data within each observer as well as a sharp peak. Peak frequency for HS is 5.96 Hz (SD = 0.45), 5.96 Hz (SD = 0.62) for RN, and 5.31 Hz (SD = 1.02) for MW excluding 0 Hz. Peak frequencies may seem slightly higher than those reported in Experiment 2, a fact we attribute to the use of a different measure in this experiment. Most importantly, however, all observers show remarkably constant perceived-frequency rates despite considerable variations in physical stimulus frequencies. Figure 6D confirms that the apparent frequency of physical saltation, as measured for stimuli composed of grating and window moving together intermittently at one of several temporal frequencies (1.5–7.5 Hz), was counted accurately. Thus, the steady number of illusory saltations reported by observers was not an artefact inherent to difficulties either in counting cycles or in working memory capacity. Together, results of Experiments 2 and 3 provide joint evidence that the temporal rate of illusory saltation remains largely constant over a wide array of stimulus characteristics, especially, within individuals. Data therefore suggest that illusory saltation is likely mediated by cyclic processes operating in the theta rhythm band.

## Discussion

The current study presents a novel visual illusion in which a smoothly moving pattern is perceived as jumping periodically across head-centered space at rhythms in the theta-band frequency range (3–8 Hz). Properties of this new illusion are markedly different than those of previously reported examples of discretized perception operating in retinotopic space in the alpha band (8–15 Hz)^11,12^. Together with recent reports of periodic fluctuations of performance data on several behavioral tasks^16–22^, our results provide further evidence for cyclical visual processing in the theta-band rhythm regime. The vivid sense of a discretized perceptual experience provides direct evidence that theta-band processing rhythms are involved in our continuous perception of dynamic visual events.

In principle, the phenomenology of illusory saltation (exemplified by the results of Experiment 1) can be described by a simple positional reset model that has been applied to previous reports of jitter illusions^11,12^. In the case of our illusion, the dissociation between the grating and window’s positional representations should increase with the velocity difference between the two components of the stimulus, particularly if both components move in opposite directions (c.f. apparent position shift induced by motion^28,29^). The visual system should tolerate this dissociation up to a point but would need to resolve it intermittently. A periodic illusory saltation should occur at moments when the discrepancy is reset. In line with this positional reset model, we would expect illusory saltation to occur at a faster temporal rate if the window moves more rapidly than the grating. However, data from Experiments 2 and 3 indicate that the temporal rate of illusory saltation remains largely constant over a wide array of stimulus characteristics, thereby ruling out a reset model premised on the dissociation between the positional representations of the grating and the window as a phenomenal level. Instead, data suggest that illusory saltation is likely mediated by cyclic processes operating in the theta rhythm band.

To date, this slow cyclic theta-band process is interpreted as reflecting the periodicity of an attentional network distinct from fast cyclic processes for sensory integration^19,30–32^. Attention’s major role is not only to facilitate and speed up stimulus processing^33,34^ but also to bind perceptual information across different dimensions^35^, in particular between location (where/when) and feature (what), that are processed along different cortical streams^36^. It is known that reduced attention can result in feature misbinding, or “illusory conjunctions”^37^. These findings lead us to consider that the visual system binds location and feature information periodically because of the cyclic nature of attention, and that theta-cyclic process may produce illusory discrete perception observed in the present study. Given the functional duality of attention, it is also sensible that a cyclic attentional network would produce periodic modulations of signal detection performance^19,30–32^ as well as discrete percepts of continuous stimuli. Different lines of psychophysical evidence also show that attentive discrimination between dynamic visual stimuli has a temporal resolution limit of around 3–5 Hz^38–40^, thereby indicating that attention cannot operate beyond this relatively low temporal rate.

Tracking a visual target moving around a scene by means of eye movement or attention is one of the most fundamental functions of biological visual systems, and slow periodic phenomena associated with detection and perception may therefore reflect specific properties of such attentive tracking/localization processes. Accordingly, key evidence for slow periodic processing has been gathered under conditions involving spatial shifts of attention such as target detection at cued locations^18,19,30–32^, momentary localization of moving stimuli^21^, and the phenomenological appearance of a single moving pattern (present study). Many models of visual cognition assume that feature information is transferred to short-term memory through the spotlight of spatial attention^27,35^. If the movement of the spotlight is governed by a cyclic process, then it stands to reason that behavioral judgments of the target would exhibit periodical fluctuations as well. In the novel discrete motion illusion reported here, luminance motion signals bias target location away from the attentional spotlight that oscillates on and off. As a result, observers experience periodic saltations and perceive a flickering target.

Several findings have shown that theta-band periodical processes mediate tasks such as conjunction searches^41–43^ widely known to solicit attention. While our attention-based account of illusory saltation remains speculative and is not yet based on evidence where attention has been manipulated directly, we posit that illusory saltation may provide a window into attention’s function of periodically binding perceptual information.

Combined with evidence for alpha-rhythm perception reported in previous studies^11–15^, our evidence for distinct discrete theta-rhythm perception suggests that visual perception is mediated by at least two cyclic neural mechanisms. It has long been suggested that the visual system is equipped with two or three levels of neural processes dedicated to the analysis of spatiotemporal patterns in the retinal image^44,45^. It is generally believed that higher-level processes integrate information over larger spatiotemporal extents and across dimensions. On the assumption that each level of analysis involves cyclical processing, one would expect higher-level process to operate at lower temporal rates in order to accommodate the long-range cortical interactions required to integrate diverse information. Indeed, whereas discrete alpha-band perceptual phenomena such as the jitter illusion^11,12^ have been observed for luminance and color stimuli detected by low-level processes, our discrete theta-band motion illusion involves a contrast-defined spatial window whose position can only be encoded by higher-level processes^46^. Recent reports of alpha-band neural correlates of temporal integration and/or cross-modal interaction^47,48^ seemingly contradict this low- vs. high-level dichotomy, but the perceptual task in such reports only requires the processing of first-order stimulus attributes. In contrast, higher-level processes, including those encoding 2nd-order stimulus attributes, are generally under strong attentional influence^49,50^, and it is therefore sensible that the slow cyclic nature of the theta-band process also involves attentional components.

Illusory alpha-rhythm jitter percepts have been causally linked to neural oscillations inherent to individuals^51^, and a similar functional relation can be imagined between apparent saltation rhythms and their underlying neural signals. Theta-band oscillations may reflect the physiological workings of slow cyclic processes that involve, on the one hand, guiding one’s attention and, on the other hand, maintaining the temporal continuity of perception. The present study has revealed no such associations, but it remains an open question to be addressed in future studies.

### Data availability

The dataset is available online on figshare public repository.

## Electronic supplementary material

Supporting Information

Movie S1

Movie S2

Movie S3

Movie S4

Movie S5
