# Supplementary material for: Discretized Theta-Rhythm Perception Revealed by Moving Stimuli
Source: Sci Rep. 2018 Apr 9;8:5682. doi: 10.1038/s41598-018-24131-6 (PMC5890248; doi:10.1038/s41598-018-24131-6)
Supplement: Supplementary file 1 — Supporting Information [file 41598_2018_24131_MOESM1_ESM.docx]

Discretized Theta-Rhythm Perception Revealed by Moving Stimuli

Ryohei Nakayama^1^*, Isamu Motoyoshi^1^, Takao Sato^2^

^1^Department of Life Sciences, The University of Tokyo, 3-8-1 Komaba, Meguro-ku, Tokyo, 153-8902, Japan

^2^Department of Comprehensive Psychology, Ritsumeikan University, 2-150 Iwakura-cho, Ibaraki, Osaka, 567-8570, Japan

*Correspondence to: [ryouhei.nakayama@gmail.com](mailto:ryouhei.nakayama@gmail.com)

Supplementary movies: 5

Supporting Information

Stimulus movies


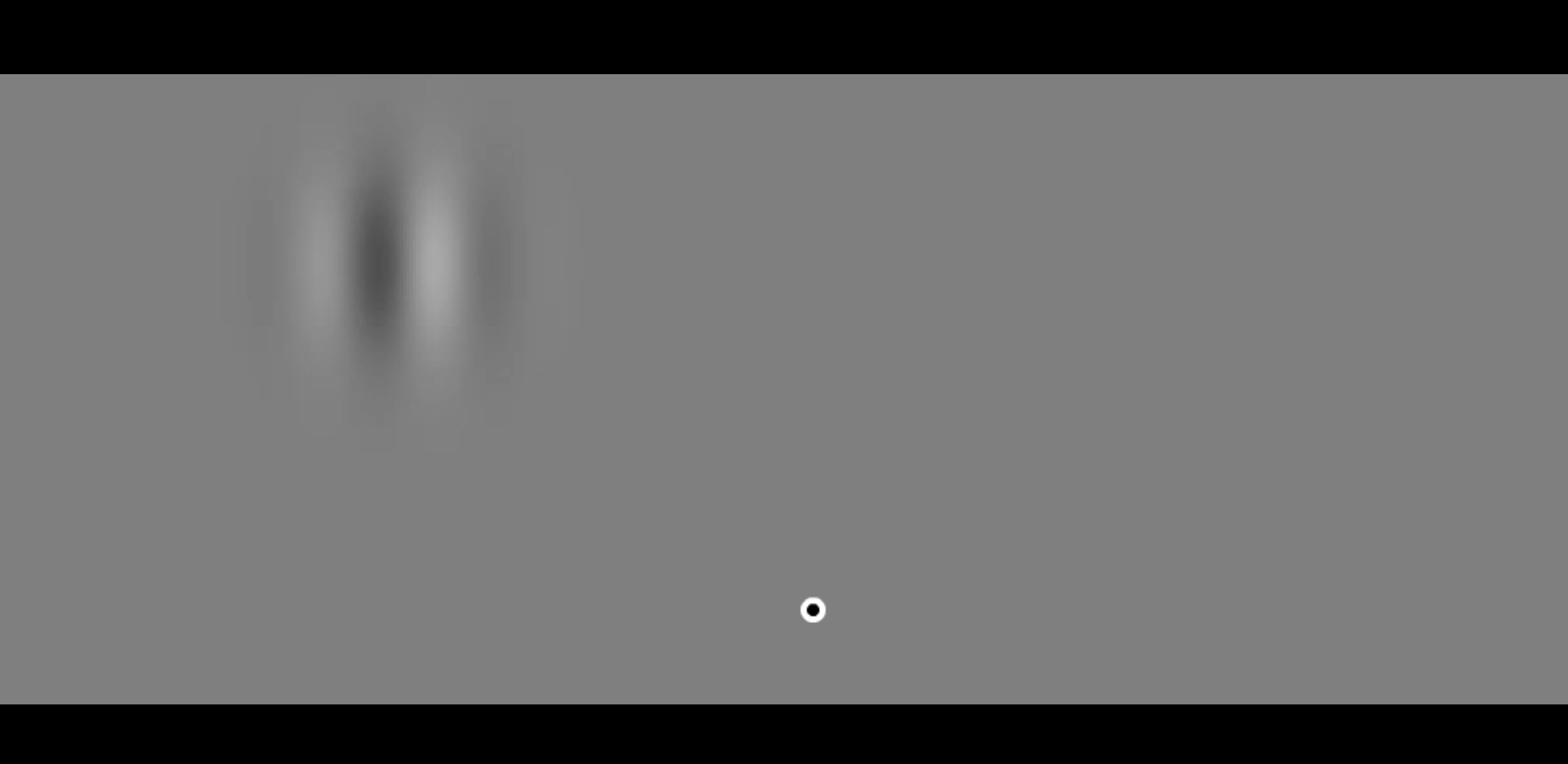


Movie S1. The spatial window of a Gabor pattern moves continuously while the carrier grating drifts in the opposite direction. In such displays, the whole stimulus appears as a slow succession of stationary snapshots (illusory saltation).

Movie S2. The spatial window of a Gabor pattern moves continuously while the carrier grating remains stationary. In such displays, the whole stimulus appears as a slow succession of stationary snapshots (illusory saltation).

Movie S3. The carrier grating drifts in the same direction as the spatial window but moves at a slower speed. In such displays, no saltation is perceived (continuous motion).

Movie S4. The carrier grating drifts in tandem with the spatial window. In such displays, no saltation is perceived (continuous motion).

Movie S5. The carrier grating drifts in the same direction as the spatial window but moves at a higher speed. In such displays, little or no saltation is perceived. (The ratings were found to be around “3” at their peaks in Figure 4a, as weak as those for gratings drifting within a static window. Taking into a consideration that phenomena like illusory saltation have never been reported in regard to perception of such stimuli, these ratings should only mean that moving stimuli looked not so smooth compared to continuous motion consistent between the grating and the window.)
